# *Atg7* deficiency in microglia drives an altered transcriptomic profile associated with an impaired neuroinflammatory response

**DOI:** 10.1186/s13041-021-00794-7

**Published:** 2021-06-03

**Authors:** Lara Friess, Mathilde Cheray, Lily Keane, Kathleen Grabert, Bertrand Joseph

**Affiliations:** Toxicology Unit, Institute of Environmental Medicine, Karolinska Institutet, Stockholm, Sweden

**Keywords:** *Atg7*, Inflammatory response, Microglia, Neurotoxicity, NF-κB, Transcriptome

## Abstract

Microglia, resident immunocompetent cells of the central nervous system, can display a range of reaction states and thereby exhibit distinct biological functions across development, adulthood and under disease conditions. Distinct gene expression profiles are reported to define each of these microglial reaction states. Hence, the identification of modulators of selective microglial transcriptomic signature, which have the potential to regulate unique microglial function has gained interest. Here, we report the identification of ATG7 (Autophagy-related 7) as a selective modulator of an NF-κB-dependent transcriptional program controlling the pro-inflammatory response of microglia. We also uncover that microglial *Atg7*-deficiency was associated with reduced microglia-mediated neurotoxicity, and thus a loss of biological function associated with the pro-inflammatory microglial reactive state. Further, we show that *Atg7*-deficiency in microglia did not impact on their ability to respond to alternative stimulus, such as one driving them towards an anti-inflammatory/tumor supportive phenotype. The identification of distinct regulators, such as *Atg7*, controlling specific microglial transcriptional programs could lead to developing novel therapeutic strategies aiming to manipulate selected microglial phenotypes, instead of the whole microglial population with is associated with several pitfalls.

**Supplementary Information:**

The online version contains supplementary material available at 10.1186/s13041-021-00794-7.

## Introduction

Microglia are the resident immunocompetent cells of the central nervous system, reported to populate the brain during early embryonic development and to derive from myeloid precursors originating from the yolk sac in mouse [1]. Accumulating data demonstrates a vast array of functions for these myeloid cells across development, adulthood and under disease conditions (reviewed in [2, 3]). Adequate microglial functions are crucial throughout life for neurogenesis, neuronal circuit shaping, vascular formation and remodeling, and overall maintenance of brain homeostasis (reviewed in [4, 5]). However, microglia can also become over-activated or deregulated in the context of brain injury, neurodegenerative and proliferative disorders, and thereby could contribute to disease severity. Undeniably, activation of microglia and inflammation-mediated neurotoxicity are suggested to play a decisive role in the pathogenesis of several neurodegenerative disorders like Alzheimer’s disease, Parkinson’s disease, Huntington’s disease, as well as amyotrophic lateral sclerosis [6, 7]. Uncontrolled microglia will also lead to increased tumor cell migration and invasion in the case of primary and metastatic brain tumors [8–10]. Thus, it is now recognized that microglia can display a wide range of reaction states based on stimuli they perceive and thereby exert distinct, sometime contrasting, functions. Another consolidating view in the field is that the numerous functions of microglia would be fulfilled through their reaction toward multiple phenotypes, each associated with a distinct transcriptomic signature (reviewed in [11, 12]). In fact, the number of distinct gene expression profiles used to define either various microglial reaction states, or transcriptomes associated to diseases are accumulating [13–23]. As a result, identifying potential modulators of these distinct microglial transcriptomic profiles, which are both associated with unique functions and linked to diseases, has gained increased interest in the microglia field. We brought our attention to ATG7 (Autophagy related 7) encoded by the *Atg7* gene, considering reports indicating involvement of this protein in the regulation of transcription as well as cell phenotypes. Of interest, microglial *Atg7* deficiency has been reported to impact on several animal models of diseases, including autism spectrum disorders [24], multiple sclerosis [25], Alzheimer’s disease [26] and seizures [27]. The encoded protein is an E1-like activating enzyme, which is known for its essential role in macroautophagy (hereafter referred to as autophagy), where it activates ATG12 for its conjugation with ATG5 as well as the MAP1LC3/ATG8 family proteins for their conjugation with phosphatidylethanolamine (reviewed in [28]). Worth a note, the expression of ATG5 in microglia is reported to not be essential to autoimmune neuroinflammation response in mice [29].

However, independently of its E1-like enzymatic activity, ATG7 is reported to bind to the transcription factor p53 and thereby regulates the expression of target genes such as *CDKN1A*, which encodes for the cyclin-dependent kinase (CDK) inhibitor p21. Hence, ATG7 is thought to modulate p53-dependent cell cycle control [30]. ATG7 is also suggested to bind to another transcription factor, FOXO1 (Forkhead box protein O1), when present in the cytoplasm and is acetylated [31]. In bladder cancer cells ATG7 is reported to indirectly repress the expression of *FOXO1,* which controls the expression of *CDKN1B* gene encoding for a second CDK inhibitor p27 [32]. One additional example of a transcription factor proposed to be regulated by ATG7 and of particular interest for the present study, is nuclear factor of kappa B (NF-κB). In fact, tumor necrosis factor α-induced NF-κB nuclear translocation was found to be abolished in *Atg7*-deficient [33]. Worth a note, the non-autophagy functions of the molecular players in the autophagy machinery has attracted increased attention in recent years (reviewed in [34, 35]). In the present study, we investigated whether *Atg7* deficiency could impact the ability of microglial cells to respond to stimuli in their microenvironment and to acquire distinct activation states associated with unique biological functions.

## Materials and methods

### Reagents and antibodies

Reagents used in this study include bafilomycin A_1_ (BafA_1_, Santa Cruz Biotechnology, sc-201550), DAPI (Invitrogen, P36931), Hoechst 33342 (Invitrogen, H3570), IL-4 (Peprotech, 214-14), LPS (Sigma, L2654), Phalloidin Alexa Fluor™ 594 (Invitrogen, A12381), ProLong Gold antifade mountant with DAPI (Life technologies, P36935) and Torin1 (Tocris, 4247). Primary antibodies were used according to manufacturer’s instructions. A list of primary and secondary antibodies used in this study is shown in Table 1.

**Table 1:** List of antibodies

Antibodies	Companies
Primary antibodies	
ACTB, mouse monoclonal antibody	Sigma-Aldrich (A-3853)
APOE, rabbit monoclonal antibody	Abcam (ab183597)
ARG1, rabbit polyclonal antibody	Abcam (ab91279)
ATG7, rabbit polyclonal antibody	Abcam (ab133528)
GAPDH, mouse monoclonal antibody	Millipore (CB1001)
LAMIN A/C, mouse monoclonal antibody	Sigma-Aldrich (SAB4200236)
LC3B, rabbit polyclonal antibody	Sigma-Aldrich (L7543)
NOS2, rabbit polyclonal antibody	Cell signaling (13120)
P65, NF-κB subunit, rabbit polyclonal antibody	Santa Cruz (sc-372)
Secondary antibodies	
IRDye^®^ 800CW Goat anti-Mouse IgG	Li-COR (926-32210)
IRDye^®^ 680RD Goat anti-Rabbit IgG	Li-COR (926-68071)
Donkey anti-Rabbit IgG, Alexa Fluor 488	Invitrogen (A21206)

List of primary and secondary antibodies used in this study, the respective companies and the catalogue numbers

### Generation of *Atg7* knockdown BV2 cells

The sh*Atg7* BV2 and shCtrl BV2 cell lines were a generous gift from Dr. Seong-Woon Yu (Daegu Gyeongbuk Institute of Science and Technology, Republic of Korea) and their generation has been previously described [36]. Briefly, shCtrl and sh*Atg7* lentiviruses (TRCN0000092164, Sigma-Aldrich) were produced following published protocol [37]. BV2 cells were infected with the lentiviruses in the presence of hexadimethrine bromide (8 µg/ml) for 24 h and then the medium was replaced with fresh culture medium. After 72 h, cells were selected using puromycin (8 µg/ml) for 2–3 days, and puromycin-resistant cells were re-seeded and grown in fresh culture medium.

### Cell culture and treatment

BV2 shCtrl and sh*Atg7* cells, glioma C6 (ATCC^®^ CCL-107™) and neuronal MN9D (gift from Dr. Alfred Heller, University of Chicago, USA) rodent cell lines have been used in this study. BV2 and C6 cells were cultured in complete DMEM Glutamax (Gibco, 61965059) with 10% fetal bovine serum (Gibco, 10270-106, LOT 42F1490K) and 1% penicillin/streptomycin (Gibco, 15140122). MN9D cells were cultured in DMEM/F12 (Gibco, 31330038) with 10% fetal bovine serum and 1% penicillin/streptomycin. Cells were regularly tested with LookOut mycoplasma detection kit (Sigma, MP0035). BV2 cells were treated with either 100 ng/ml LPS (likewise MN9D cells, in the coculture experiment), 10 ng/ml IL-4, or 250 nM Torin1 for the indicated time points. As control, cells were treated with the respective reagent’s solvent. BV2 cells were starved using Earle’s Balanced Salt Solution (EBSS medium, Gibco, 24010043). Co-treatment with 40 nM BafA_1_, 2 h before sample collection, was used to block autophagic flux.

### Immunofluorescence and confocal microscopy

BV2 cells, seeded onto coverslips, were fixed with paraformaldehyde (PFA, Sigma, F8775) with a final concentration of 4% for 15 min. They were washed with 0,1% Triton X (Sigma, X100) in PBS (Santa Cruz Biotechnologies, sc-362299) and incubated with a blocking solution containing 10% donkey serum (Jackson Immuno Research, 017-000-121), diluted in PBS/Triton X. Primary ATG7 antibody, diluted 1:400 in blocking solution, was incubated overnight. Secondary antibody diluted 1:400 in PBS/Triton X, and Phalloidin Alexa Fluor™ 594 diluted 1:200, were incubated for 1 h at room temperature. Finally, nuclei were counter-stained using 1 µg/ml Hoechst 33342 dye and coverslips were mounted onto glass slides using Fluoromount-G mounting medium (Southern Biotechnology, 0100-01). Images were acquired using the Zeiss LSM800 confocal laser scanning microscope and analyzed with ZEN software (Black edition 3.0, Zeiss).

### Subcellular fractionation

Cell lysis and extraction of the cytoplasmic and nuclear fractions was performed using the NE-PER™ Nuclear and Cytoplasmic Extraction kit (Thermo Scientific, 78835) according to the manufacturer’s instructions. The resulting fractions were then mixed with 5× Laemmli buffer (62.5 mM Tris–HCl, pH 6.8 (Sigma, T6066), 2% sodium dodecyl sulfate (Sigma, L4509), 10% glycerol (Sigma, G5516), 5% beta-mercaptoethanol (Sigma, 63689), 0.02% bromophenol blue (Bio Rad, 1610404)) and separated by SDS-polyacrylamide gel electrophoresis.

### Protein analysis by Western blot

Cells were collected directly in 2,5× Laemmli buffer using a cell scraper. Samples were sonicated (Diagenode, Bioruptor Pico) and boiled, proteins were then separated by SDS–polyacrylamide gel electrophoresis and blotted onto 0.2 μm or 0.45 μm pore-size nitrocellulose membranes (Bio Rad, 1620112 and 1620115) using the Mini Trans-Blot wet transfer system (Bio Rad, 1703989). Membranes were blocked with 0.1% Tween 20 (Sigma-Aldrich, P1379) and 5% milk (Semper, 31909) in PBS (Santa Cruz Biotechnology, sc-362299) and incubated overnight at 4 °C with indicated primary antibodies (listed in Table 1) following manufacturer’s instructions. Membranes were incubated with RDye^®^ secondary antibodies according to the manufacturer’s instructions. Results were visualized using the Odyssey CLx infrared imaging system (LI-COR Biosciences, Lincoln, NE, USA) with the software Image Studio Lite, version 5.2 (LI-COR Biosciences). All targeted proteins of interest were normalized to the selected housekeeping protein Actin (ACTB), LAMIN C or GAPDH and intensity of the bands was quantified using ImageJ software.

### RNA isolation and RT-qPCR

RNA was isolated using the RNeasy Plus Mini kits (Qiagen, 74134). cDNA was synthesized using Superscript IV, Oligo dT and dNTP (Invitrogen, 18090010). RT-qPCR was performed with the StepOne plus instrument (Applied Biosystems) using the SYBR Green master mix (Applied Biosystems, 4385617) and predesigned primers (KiCqStart Primers, Sigma, listed in Table 2). Relative gene expression levels were normalized to β-actin mRNA expression in each sample using the ΔΔCT method.

**Table 2:** List of RT-qPCR primers

Genes	Forward primer	Reverse primer
RT-qPCR primers		
* Actb*	GATGTATGAAGGCTTTGGTC	TGTGCACTTTTATTGGTCTC
* Apoe*	ACCTGATGGAGAAGATACAG	GATATGGATGTTGTTGCAGG
* Arg1*	GGAGACCACAGTCTGGCAGTTGGA	GGACACAGGTTGCCCATGC
* Atg7*	CTGTTCACCCAAAGTTCTTG	TCTAAGAAGGAATGTGAGGAG
* Nfkb1*	AGGACATGGGATTTCAGGA	GGAGGTGGATGATGGCTAA
* Nfkb2*	TCAAGATCTGTAACTATGAGGG	TTCTTCTTGGTTACATGCAG
* Nos2*	GCGGAGTGACGGCAAACAT	AGGTCGATGCACAACTGGG
* Rel*	ATACCTGCCAGATGAAAAAG	TCAGTAAAGTGACCACAATC
* Rela*	GCTCCTAAGGTGCTGACA	ACCTCCGAAAGCGAGATA
* Relb*	CGGATTTGCCGAATCAAC	CGGATATGTCCTCTTTTTGC

List of primer sequences used in this study. All the sequences are given 5ʹ to 3ʹ

### Library preparation and RNA-Seq

2 ng of total RNA was used to generate cDNA for the samples using the Smart seq V4 Clontech reagents. Libraries were prepared with the Illumina Nextera XT protocol. The library pools underwent cluster generation on an Illumina cBot and the clustered flow cell was sequencing on the IlluminaHiSeq 2000 instrument to generate 50 bp SR. The raw sequencing reads were processed through CASAVA for FASTQ conversion and demultiplexing.

### RNA-Seq data and computational analysis

Bcl files were converted and demultiplexed to fastq using the bcl2fastq v2.20.0.422 program. STAR v2.7.5b was used to index the mouse reference genome (mm10/GRCm38) and align the resulting fastq files. Mapped reads were then counted in annotated exons using featureCounts v1.5.1. The annotations and reference genome were obtained from Ensembl. The count table from featureCounts was imported into R/Bioconductor and differential gene expression was performed using the EdgeR v3.30.3 package and its general linear model pipeline. For the gene expression analysis genes that had 1 count per million in 3 or more samples were used and normalized using TMM normalization.

Differentially expressed genes with significant (< 0.05) FDR were used for all further analysis. The genes shown in the heatmap are genes that were found significantly regulated in any of the comparisons. Each gene (row) is standardized (z) to mean = 0 and sd = 1 and then clustered by hierarchical clustering. Volcano plot as well as bar graphs were generated using Excel (Microsoft Office v365). The analysis of Gene Ontology (GO) terms was performed using the bioinformatics database Metascape (https://metascape.org/, [38]). Gene lists of interest were imported and the resulting enrichment map file then adjusted for visual preferences using the Cytoscape software (v3.7.1, www.cytoscape.org; [39]). The Enrichment Map (www.baderlab.org/Software/EnrichmentMap) plug in was used as a visualization tool to produce a network graph of over-represented GO terms. Enrichment was based on an FDR of maximum 0.05. Nodes represent enriched GO terms and node size correlates with the number of genes included in a specific GO term. Edges indicate the degree in overlap between nodes. In addition to the enrichment maps for all significant differentially expressed genes, enrichment analysis was performed on a subset of those genes that overlap with immunologic signature genes. Gene lists for each condition were compared to the Gene Set Enrichment Analysis (GSEA) gene set collection C7: immunologic signature gene sets (http://www.gsea-msigdb.org/gsea/msigdb/collections.jsp#C7%EF%BC%8CC7, accessed 06.05.2021) and the overlap of both sets subsequently used to create enrichment maps as described above. Transcriptional regulatory relationships unraveled by sentence-based text mining (TRRUST), a bioinformatics tool provided through the TRRUST website (www.grnpedia.org/trrust; [40]), was used to show transcription factors associated with our gene lists of interest, using a cut-off of 1.5 for the total fold change. KEGG pathway analysis was performed on gene sets of interest using the EnrichR database (https://maayanlab.cloud/Enrichr/#, [41]). The Venn diagram is based on differentially expressed gene lists for each LPS-stimulus condition for the BV2 shCtrl and sh*Atg7*, respectively. The diagram was created using the Venny tool (v2.1, https://bioinfogp.cnb.csic.es/tools/venny/ [42]).

### Chromatin immunoprecipitation (ChIP)

NF-κB P65 subunit ChIP experiments were done using the iDeal ChIP-qPCR kit (Diagenode, C01010180) according to the manufacturer’s instructions. Briefly, after cell cross-linking in 1% formaldehyde and cell lysis of 16.10^6^ cells per condition, chromatin shearing was done with a Bioruptor^®^ Pico sonicator (Diagenode) (8 cycles, 30 s ON/30 s OFF). Then, each chromatin immunoprecipitation was done overnight using NF-κB P65 ChIP grade antibody (Cell Signaling #8242) or normal rabbit IgG antibody (R&D, AB-105-C) used as control. Purified DNA and 1% input were then analyzed by qPCR using primers targeting the *Nos2* gene (Forward: 5′-GTCCCAGTTTTGAAGTGACTACG-3′; Reverse: 5′-GTTGTGACCCTGGCAGCAG-3′) as previously described [43]. Data interpretation from qPCR was done by calculation of the percentage to input and then normalized to the non-treated condition.

### Neuronal cell death assay

Microglial BV2 and dopaminergic MN9D murine cells were cultured as described. MN9D dopaminergic neuronal cells were stained with CellTracker Green CMFDA (Invitrogen, C7025) and washed, before shCtrl or sh*Atg7* BV2 microglia, respectively, were plated as a direct co-culture. The cells were given at least 4 h to attach to the plates and were then treated with LPS (100 ng/ml) and incubated for an additional 24 h. They were stained with 0.1 mg/ml Hoechst and counted for quantification of neuronal cell death.

### Cytofluorometric analysis of ROS

Intracellular ROS was detected using a CM-H2DCFDA kit (General Oxidative Stress Indicator, Invitrogen, C6827). Samples were collected in cold HBSS (Gibco, 14175095) and kept on ice until the analysis with the BD LSR2 Cell analyzer. 5 µM dye was added 20 min before the read out on site. 100 µM hydrogen peroxide (Sigma-Aldrich, H3410) for 30 min was used as a positive control. Data were analyzed using Flowjo (BD Biosciences, v10).

### Glioma migration assay

BV2 cells were seeded in medium with 10% serum, C6 glioma cells were seeded in inserts with 8 µm pores (Falcon, 353097) in medium with 5% serum. Inserts were then placed onto BV2 cells, or medium only as control, and incubated together for 6 h. C6 cells in inserts were fixed with 4% PFA and non-migrated cells removed with a swab. The remaining insert membranes were mounted onto glass slides with medium containing DAPI to be counted under the microscope.

### Statistical analyses

Statistical analyses when comparing the two cell lines shCtrl BV2 and sh*Atg7* BV2 within one treatment condition were carried out using the one-tailed Student t test, assuming homoscedastic variance. FACS data was analyzed using the Chi square test comparing the two populations either with or without LPS stimulus. All values are a mean of at least 3 independent experiments ± SEM and considered significant for *p < 0.05, **p < 0.01, ***p < 0.001, ****p < 0.0001, or n.s., not significant, for the indicated comparison.

## Results

### Reduction in *Atg7* expression levels does not prevent microglia from undergoing autophagy

**Fig. 1:**
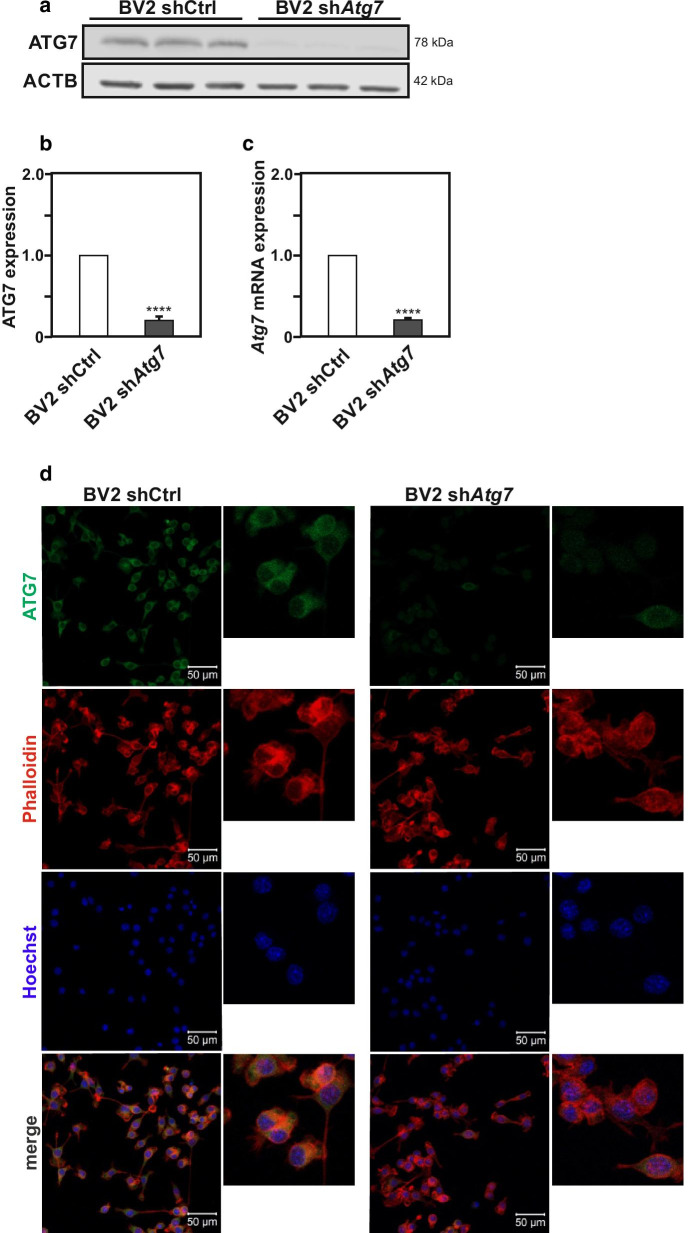
Stable knockdown of *Atg7* gene expression using small-hairpin RNA in BV2 microglia. **a** Immunoblot analysis of ATG7 protein expression levels in BV2 microglia cell lines infected with lentivirus expressing a shRNA targeting *Atg7* expression (sh*Atg7* BV2 cells), or with a control scramble shRNA (shCtrl BV2 cells). The expression of ACTB as housekeeping gene was used as loading control. **b** The graphs show the quantification for ATG7 versus ACTB protein expression ratio in sh*Atg7* BV2 cells as compared to shCtrl BV2 cells used as control. **c** Comparison of *Atg7* mRNA expression measured by RT-qPCR in sh*Atg7* BV2 cells and shCtrl BV2 cells. **d** Immunofluorescence analysis of ATG7 protein expression in sh*Atg7* BV2 cells and shCtrl BV2 cells. Chromosomal dye Hoechst is used for nuclear counterstaining. Phalloidin staining shows actin filaments (scale bar 50 µM). All values are a mean of three independent experiments ± SEM and considered significant for ****p < 0.0001

To address a potential role for *Atg7* in controlling the acquisition of different microglial reactive states, we took advantage of a microglial cell line generated with a previously established short hairpin RNA (shRNA) strategy, with a stable reduction in *Atg7* expression [36] (Fig. 1a–d). shCtrl BV2 microglia infected with the control scramble lentiviral vector exhibited robust ATG7 protein (Fig. 1a, b) as well as *Atg7* mRNA expression (Fig. 1c). In contrast, sh*Atg7* BV2 infected with the lentiviral vector driving the expression of a shRNA targeting *Atg7* gene expression, showed a significant (> 80%) knockdown of its expression, as illustrated by the immunoblot and RT-qPCR analyses of ATG7 protein and *Atg7* mRNA expression, respectively (Fig. 1a–c). Immunofluorescence analysis in shCtrl BV2 cells and sh*Atg7* BV2 cells further confirmed the robust reduction of ATG7 in sh*Atg7* cells. Nuclear counterstaining with the chromosomal dye Hoechst 33258 as well as co-labeling with Phalloidin indicated, as previously reported in other cell types ([30]; www.proteinatlas.org/ENSG00000197548-ATG7/cell), cytoplasmic as well as nuclear subcellular localization for the ATG7 protein in microglial cells (Fig. 1d).

The ability of BV2 microglia with reduced ATG7 protein expression to undergo autophagy upon exposure to an autophagy-inducing stimulus (amino acid starvation or treatment with Torin1) was examined by evaluating the conversion ratio of LC3-I to LC3-II by immunoblot analysis (Additional file 1: Figure S1a, b). As compared to shCtrl BV2 cells, sh*Atg7* BV2 cells clearly exhibited a decreased autophagic flux. However, the *Atg7* gene expression knockdown, which remained partial, did not completely abrogate the ability of these cells to respond to both autophagy stimuli. Bafilomycin A_1_ (BafA_1_) is a late inhibitor of autophagy preventing the fusion between autophagosomes and lysosomes and thus blocks LC3-II/autophagosome degradation. BafA_1_ co-treatment experiments were performed to confirm the reduction in LC3 conversion but not its absence in microglial cells with reduced ATG7 protein expression (Additional file 1: Figure S1a, b). Collectively these data indicate that the sh*Atg7* BV2 cells, despite exhibiting significant reduction in *Atg7* expression, retained the ability to respond to autophagic stimuli to at least some extend, and were not per se incapacitated for this catabolic process.

### *Atg7* deficiency in microglia results in an impaired response to lipopolysaccharide, but not to interleukin 4 stimulation

The potential impact of *Atg7* deficiency on the ability of microglia to respond to different stimuli and acquire distinct reaction states with different biological functions was investigated.

**Fig. 2:**
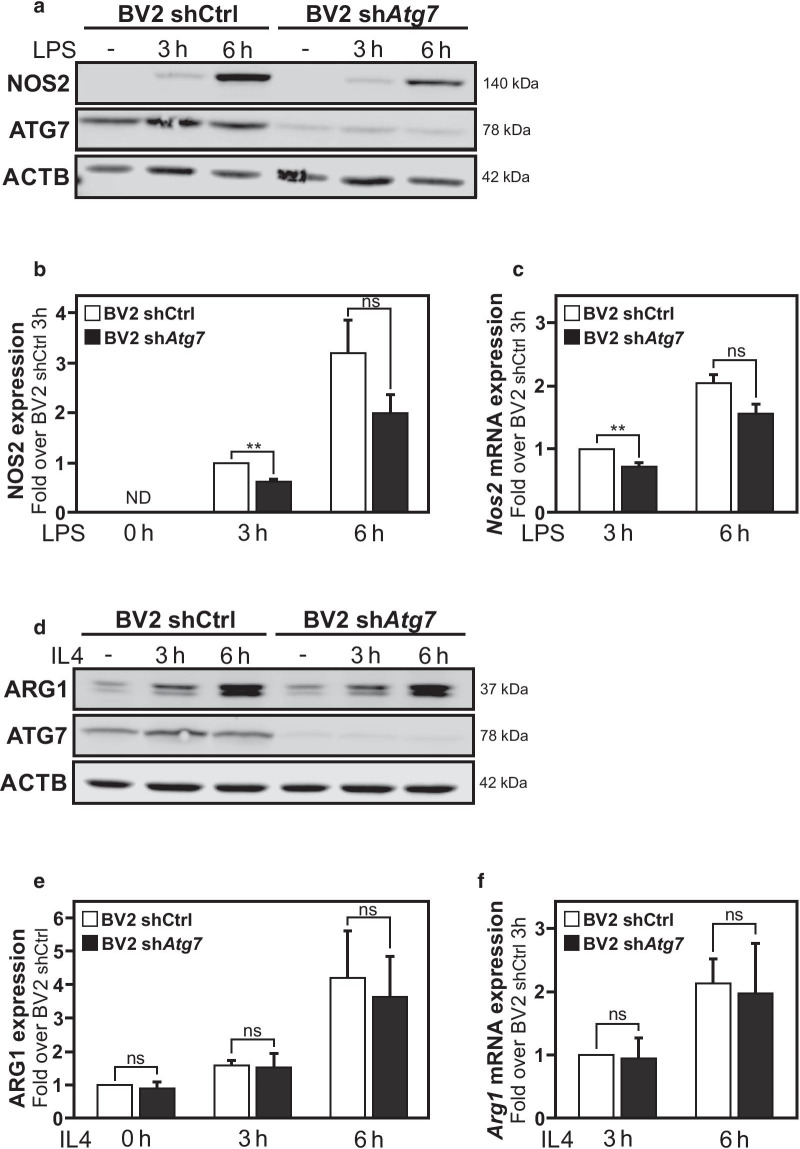
*Atg7* deficiency impairs the microglial response to lipopolysaccharide, but not interleukin 4. **a** Immunoblot analysis of NOS2 protein expression in sh*Atg7* and shCtrl BV2 cells. The expression of ACTB as housekeeping gene was used as loading control. Cells were treated with LPS (100 ng/ml) for 3 h or 6 h. **b** Graphs show the quantification for NOS2 versus ACTB protein expression and in sh*Atg7* and shCtrl BV2 cells. Values are in comparison to shCtrl BV2 after 3 h LPS. **c** Comparison of *Nos2* mRNA expression measured by RT-qPCR in sh*Atg7* and shCtrl BV2 cells. **d** Immunoblot analysis of ARG1 protein expression in sh*Atg7* and shCtrl BV2 cells. The expression of ACTB as housekeeping gene was used as loading control. Cells were treated with IL-4 (10 ng/ml) for 3 h or 6 h. **b** Graphs show the quantification for ARG1 versus ACTB protein expression and in sh*Atg7* and shCtrl BV2 cells. Values are in comparison to untreated shCtrl BV2. **c** Comparison of *Arg1* mRNA expression measured by RT-qPCR in sh*Atg7* and shCtrl BV2 cells. All values are a mean of 3 (**b**, **f**) or 4 (**c**, **e**) independent experiments ± SEM and considered significant for *p < 0.05, **p < 0.01. n.s., not significant for the indicated comparison

Bacterial endotoxin lipopolysaccharide (LPS), a toll-like receptor 4 (TLR4) ligand, is one of the most commonly used stimuli to induce a pro-inflammatory response in microglia in vitro and in animal models of neuroinflammation [6, 44]. The induction of *Nos2* gene expression, encoding for the Nitric oxide Synthase 2 (NOS2, also known as the inducible NOS, iNOS), is recognized as a consistent microglial pro-inflammatory marker in a wide range of in vitro and in vivo murine models. Therefore, induction of *Nos2*/NOS2 expression was used as a readout to assess the pro-inflammatory response of microglia carrying *Atg7* deficiency. When shCtrl BV2 cells were exposed to a LPS challenge (100 ng/ml), immunoblot analyses revealed an induction of NOS2 protein expression that was already detectable at 3 h post-treatment, and further increased at the 6 h post LPS (Fig. 2a, b). Analysis of *Nos2* mRNA levels by RT-qPCR revealed that the increased NOS2 protein expression was the result of transcriptional regulation of the *Nos2* gene in these cells (Fig. 2c). However, the induction of *Nos2* mRNA as well as NOS2 protein expression was significantly lower in LPS-treated sh*Atg7* BV2 (Fig. 2a–c). These data indicate that microglial *Atg*7 deficiency affects the ability of these immune cells to acquire a pro-inflammatory activation state. Cells that are pre-treated with BafA_1_ before LPS treatment, inhibiting the autophagic flux by decreasing autophagosome–lysosome fusion, are unable to recapitulate this reduced *Nos2* expression (Additional file 1: Figure S1c).

To determine whether the observed microglial impairment was restricted to their response to a pro-inflammatory stimulus or a more general inhibition of their ability to acquire reactive states, the effect of a non-inflammatory stimulus, i.e. interleukin 4 (IL-4) on these cells was explored. IL-4 is considered a typical regulatory cytokine that may contribute to brain repair by promoting an alternative activation state of microglia exerting protective anti-inflammatory functions. With some limitations, arginase-1 (ARG1) is currently used as a marker to identify microglia polarized toward this anti-inflammatory protective, alternative phenotype. In fact, primary microglia and microglia cell lines, including BV2 cells, are all responsive to IL-4 treatment (reviewed in [45]). The IL-4 induced microglial phenotype shares some similarity with the glioblastoma (IL-4 secreting cells)-induced tumor-supportive microglial phenotype, including the increased expression of *Arg1* gene [8]. Remarkably, in contrast to the observed differential responses to an LPS challenge, both shCtrl BV2 cells and sh*Atg7* BV2 cells treated with IL-4 (10 ng/ml) independently of their *Atg7* status, responded similarly to this stimulus. This result was illustrated by the equivalent increase in *Arg1* gene expression, measured by RT-qPCR, as well as protein expression, measured by immunoblot analysis (Fig. 2d–f). Hence, these data advocate for that reduced ATG7 protein expression in microglia impacts on the ability of these cells to respond to an inflammogen and acquire a pro-inflammatory state, but not on their ability to respond to an alternative stimulus, such as one driving them towards an anti-inflammatory phenotype.

### Microglial *Atg7* deficiency drives an altered transcriptomic profile with reduced expression of immune response-related genes

The acquisition of distinct microglial activation states, including the pro-inflammatory phenotype, is reported to firmly rely on transcriptional and associated epigenetic programs (reviewed in [12, 46–48]). Hence, the observed reduced capability of *Atg7-*deficient BV2 microglia to respond to an LPS challenge led us to investigate whether *Atg7* deficiency in microglia per se could translate into alterations in their transcriptome, i.e. their gene expression profile. To assess whether *Atg7*-deficient microglia are characterized by an individual gene expression signature, their global transcriptome was compared to that of the control microglia. For this purpose, three independent biological replicates with sh*Atg7* BV2 microglia and shCtrl BV2 microglia we collected for high-throughput RNA sequencing (RNA-Seq) analysis.

**Fig. 3:**
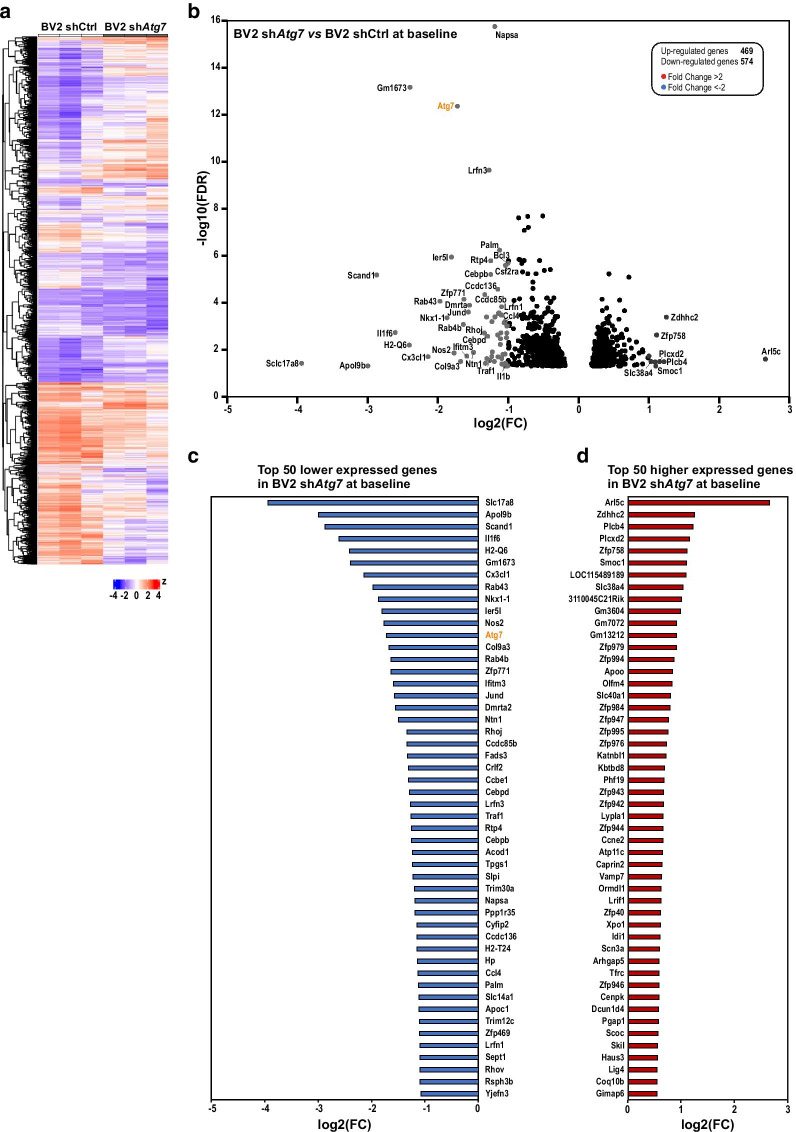
*Atg7* deficiency drives an altered transcriptomic profile in microglia. **a** Heat map of up- and downregulated genes comparing three independent replicates each of the two cell lines shCtrl and sh*Atg7* BV2 in unstimulated condition. **b** Volcano plot displaying the fold change in log2 plotted against the FDR in negative log10 in untreated condition. Blue dots represent less expressed genes in sh*Atg7* BV2 cells with a total fold change of maximally - 2, red dots represent genes that are higher expressed in sh*Atg7* BV2 cells with a total fold change of at least 2, respectively. Differential gene expression analysis revealed a total of 469 genes higher expressed, and 574 lower expressed in sh*Atg7* BV2 compared to shCtrl BV2 at baseline. Graphs show the 50 genes with the lowest (**c**) fold change and the highest (**d**) fold change in sh*Atg7* cells compared to shCtrl BV2 in untreated condition. *Atg7* is highlighted in orange with a negative log2 fold change of 1.724

RNA-Seq data revealed that the *Atg7*-deficient microglia possess a unique and distinct transcriptomic profile. Based on these genome-wide gene expression analyses, 469 genes were found to be significantly up- and 574 to be down-regulated (p values adjusted for false discovery rates, or FDR, < 0.05) compared to microglia with unaltered *Atg7* gene expression (Fig. 3a, b and Additional file 2). Even with a stringent threshold set at a twofold change (FC > 2, FC < 0.5), 63 genes and 9 genes remain respectively up- and down-regulated when the transcriptome of sh*Atg7* BV2 and shCtrl BV2 cells were compared at baseline (Fig. 3b). The top 50 genes, including *Atg7*, which exhibited lower expression in sh*Atg7* BV2 cells as compared to shCtrl BV2 cells are depicted in Fig. 3c. The top 50 genes, with higher expression in sh*Atg7* BV2 cells as compared to shCtrl BV2 cells, are presented in Fig. 3d.

**Fig. 4:**
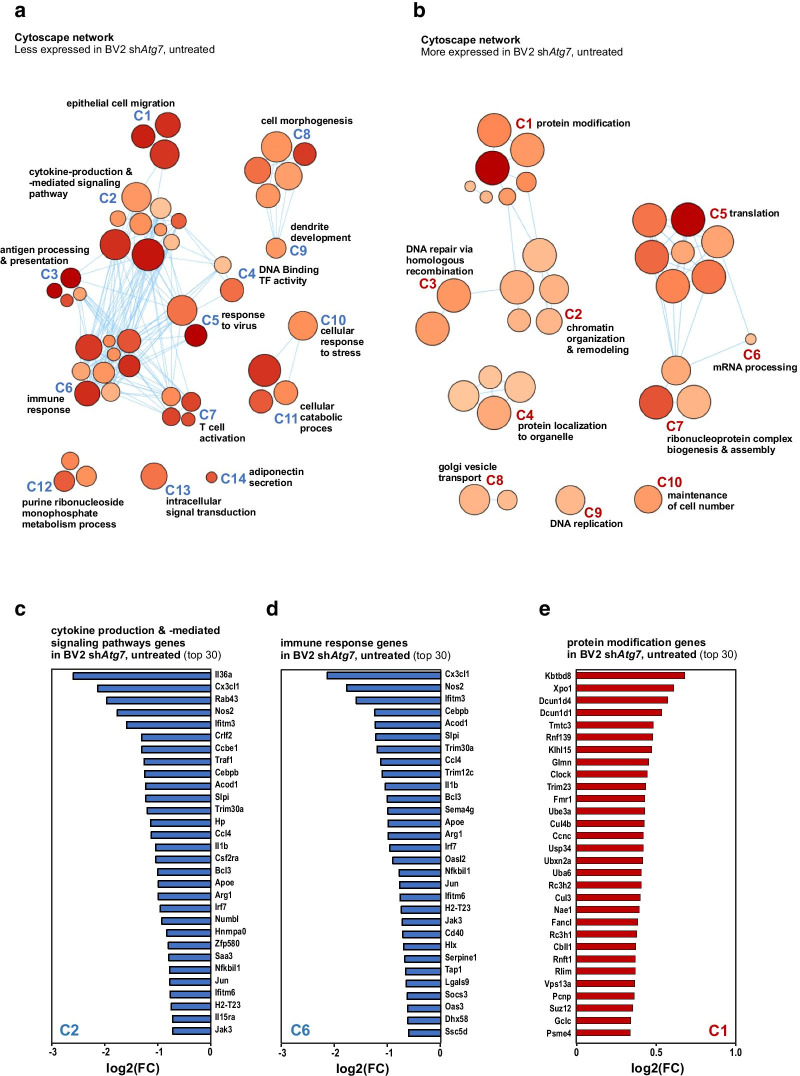
Gene Ontology network analysis reveals reduced expression of immune response-related genes in *Atg7*-deficient microglia. **a**,** b** Network analysis of enriched GO term clusters generated from differentially expressed genes with significant FDR (< 0.05). Nodes represent GO terms, clusters are nodes grouped based on similarity. Node size corresponds to number of genes. Node color corresponds to the significance of correlation, where the darker the color is, the smaller the FDR values gets. Lines represent the number of genes overlapping between nodes. Less expressed genes (**a**) versus more expressed (**b**) in sh*Atg7* BV2 as compared to shCtrl BV2 at baseline. **c**–**e** Graphs show the top 30 genes represented in the GO term clusters: **c** C2 cytokine production and cytokine-mediated signaling pathway; **d** C6 immune response and **e** C1 protein modification related genes (additional information about the clusters are available in Additional file 4)

The visualisation of gene ontology (GO) term analysis using an enrichment map showed that genes with a lower expression in sh*Atg7* BV2 cells are associated with biological processes ranging from the regulation of immune and inflammatory response, immune cell communication, to cell migration (Fig. 4a, c and d). On the other hand, Cytoscape network analysis indicated GO enrichment of higher expressed genes in sh*Atg7* BV2 for biological processes associated with metabolism, regulation of gene expression as well as protein modification (Fig. 4b and e).

**Fig. 5:**
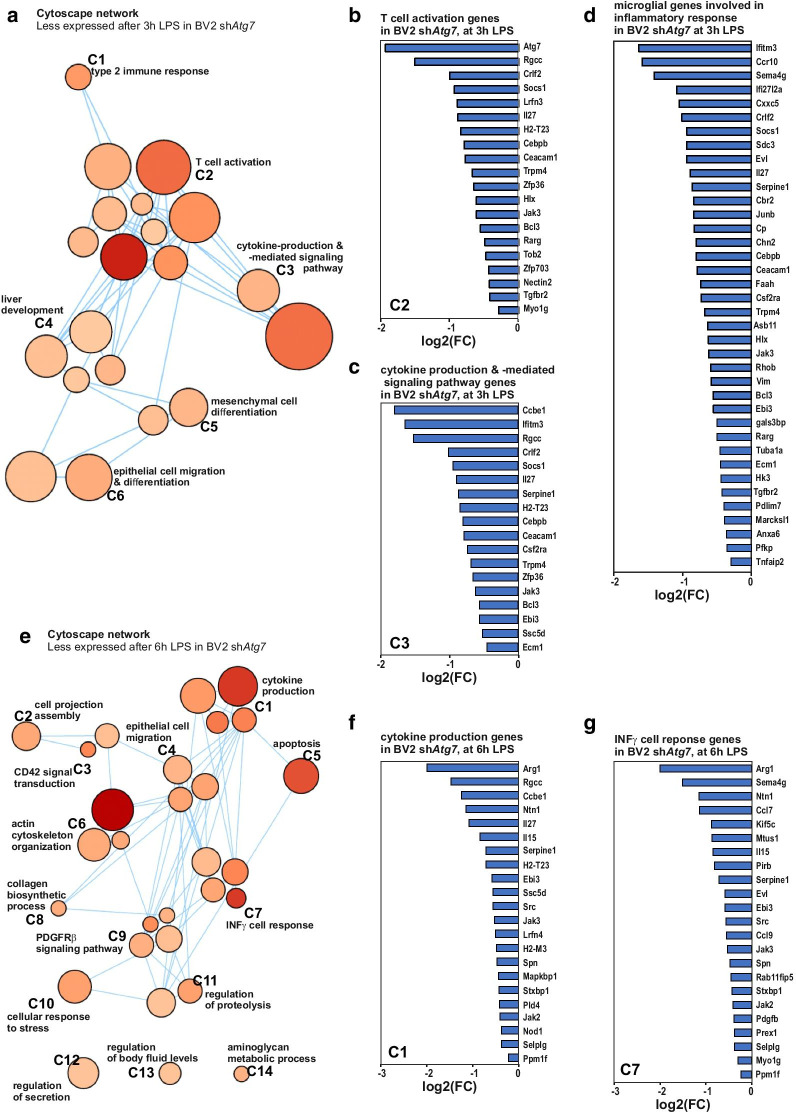
*Atg7-*deficient microglia show an altered transcriptional response to a LPS challenge. Microglial cells, i.e. sh*Atg7* and shCtrl BV2 cells were treated with LPS (100 ng/ml) for 3 h (**a**–**d**) or 6 h (**e**–**g**). Network analysis of enriched GO term clusters generated from differentially expressed genes with significant FDR (< 0.05) that are less expressed in sh*Atg7* BV2 as compared to shCtrl BV2 at 3 h (**a**) or 6 h (**e**) post-treatment with LPS are depicted. Nodes represent GO terms, clusters are nodes grouped based on similarity. Node size corresponds to number of genes. Node color corresponds to the significance of correlation, where the darker the color is, the smaller the FDR values gets. Lines represent the number of genes overlapping between nodes. Graphs show the downregulated genes represented in the GO term clusters: **b** C2 response cell activation, and **c** C3 cytokine production and cytokine-mediated signaling pathway related genes in sh*Atg7* BV2 at 3 h LPS, and **f** C1 cytokine production and **g** C7 interferon gamma (INFγ) cell response related genes in sh*Atg7* BV2 at 6 h LPS, respectively (additional information about the clusters are available in Additional file 4). **d** Graph shows the expression of genes reported in literature to be involved in the inflammatory response of microglial cells. Genes are differentially expressed at 3 h in LPS-treated sh*Atg7* BV2 microglia, as compared to the 3 h LPS-treated shCtrl BV2 microglia

**Fig. 6:**
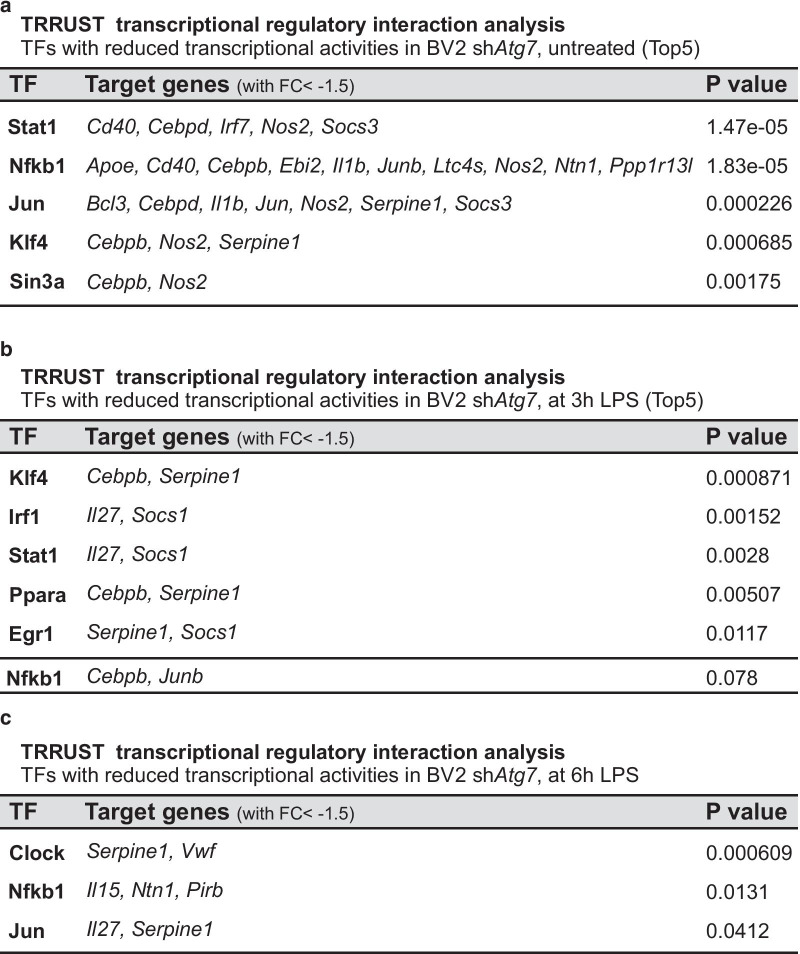
Microglial *Atg7* deficiency is associated with reduced NF-κB-dependent signaling. **a** TRRUST analysis, which allows the identification of transcription factor potentially involved in cell response based on the expression of their target genes, revealed NFKB1 as transcription factor candidate negatively affected by the microglial *Atg7* knockdown. Input genes used exhibited lower expression in sh*Atg7* as compared to shCtrl BV2 (FC < 1.5 and FDR < 0.05), significance of association is shown with the respective p value. **b**, **c** similar TRRUST-based analyses performed at 3 h (**b**) and 6 h (**c**) post LPS-treatment likewise indicated a significant negative impact on the transcriptional activity of NFKB1 at 6 h post LPS in sh*Atg7* BV2 cells as compared to shCtrl BV2 cells

The transcriptional responses of sh*Atg7* BV2 microglia versus shCtrl BV2 microglia to a pro-inflammatory challenge was also monitored. For this purpose, three additional independent biological replicates with sh*Atg7* BV2 microglia and shCtrl BV2 microglia treated with LPS (100 ng/ml) for 3 h (Fig. 5a–d) and 6 h (Fig. 5e–g) were collected and RNA-Seq analyses performed. These additional genome wide gene expression and associated GO term analysis using an enrichment map revealed at both time points mainly negative differential gene expression associated with microglial *Atg7* deficiency. In fact, when the transcriptomes of sh*Atg7* BV2 microglia were compared to shCtrl BV2 microglia, at 3 h post-LPS exposure, 93 genes were found to be significantly down-, and 11 genes up-regulated (with FDR < 0.05). At 6 h post-treatment, 119 down-regulated and 21 up-regulated genes were reported (Additional file 2). A Venn diagram illustrating the number of differentially expressed genes in 3 h and 6 h LPS treated control and *Atg7*-deficient microglia is depicted in Additional file 1: Figure S2. The genes that were less expressed in the sh*Atg7* BV2 microglia as compared to the shCtrl BV2 microglia include many linked to an immune response. In fact, the Cytoscape network analysis at 3 and 6 h post LPS-treatment indicated GO enrichment for biological processes associated with regulation of immune- and stress responses, cytokine production, and cell differentiation (Fig. 5a–g). 38 genes, that are reported in the literature to be involved in the response of microglia to an inflammatory stimulus, were differentially expressed at a significantly lower level in *Atg7*-deficient microglia 3 h post treatment (Fig. 5d). KEGG pathway analysis comparing untreated shCtrl BV2 cells with sh*Atg7* BV2 cells, as well as their LPS-treated counterpart, further supported the finding from the Metascape analysis (Additional file 2). KEGG pathway analysis was also performed analyzing the LPS-response in sh*Atg7* BV2 separately, compared to untreated sh*Atg7* BV2 (Additional file 3). In addition to analyzing all significant differentially expressed genes in each condition, we compared our data to the GSEA dataset C7: immunologic signature gene sets and used the overlap of genes as input for a further enrichment analysis (Additional file 1: Figure S3a to d). Similar to using the full gene set as input, the analysis shows clusters associated with regulating the immune effector process, inflammatory response, or response to interferon-gamma. The full subset of overlapping genes can be found in Additional file 5. Collectively, these transcriptomic data suggest that the *Atg7*-deficient microglia hold a less immune responsive phenotype than the control cells.

### Microglial *Atg7* deficiency is associated with reduced NF-κB-dependent signaling

Transcription factors and their selective target genes are central players in transcriptional regulation and in the acquisition of transcriptional programs controlling cell phenotypes. Therefore, elucidating which transcription factor-dependent signaling may be altered in the microglia carrying an *Atg7* deficiency could increase our understanding of the regulatory circuitry underlying the observed microglia phenotype, i.e. an impaired response to pro-inflammatory stimulus. For this purpose, we took advantage of the transcription factor-target interaction database TRRUST (Transcriptional Regulatory Relationships Unraveled by Sentence-based Text mining), which allows for the identification of transcription factors potentially involved in cellular responses based on the expression of their target genes. The current version of TRRUST contains 6552 regulatory relationships of 828 mouse transcription factors with their target genes (www.grnpedia.org/trrust; [40]). The 206 genes found to exhibit lower expression at baseline (FC < 1.5 and FDR < 0.05) in sh*Atg7* BV2 cells as compared to shCtrl BV2 cells were used for the TRRUST analysis. NFKB1 (nuclear factor of kappa B Subunit 1, p105), as a transcription factor candidate negatively affected by the microglial *Atg7* knockdown, exhibited the highest number of overlapped target genes (*Apoe*, *Cd40*, *Cebpb*, *Ebi2*, *Il1b*, *Junb*, *Ltc4s*, *Nos2*, *Ntn1*, *Ppp1r13l*) as well as robust statistical significance (p value = 0.000018) (Fig. 6a). Reduced expression of *Nos2*/NOS2, an established microglial proinflammatory marker, in sh*Atg7* BV2 microglia, as compared to shCtrl BV2 microglia as already been confirmed at mRNA level using qRT-PCR and protein level using immunoblot analysis (Fig. 2a–c). The validation was extended to *Apoe*/APOE*,* whose gene expression as *Nos2*, was found to be significantly decreased in sh*Atg7* BV2 microglia, as compared to shCtrl BV2 microglia, is a known-target gene for *Nfkb1* regulation, and whose expression is associated to a microglial reactive state linked to neuroinflammation [23, 49] (Additional file 1: Figure S4a–c).

The *Nfkb1* gene encodes a 105 kDa protein which can undergo processing to generate the 50 kDa DNA binding subunit p50 of the NF-kappaB (NF-κB) protein complex (reviewed in [50]). NF-κB is a ubiquitously expressed transcription factor regulating the expression of over 500 genes, many of which are implicated in inflammation-related responses (reviewed in [51]). Furthermore, similar TRRUST-based analyses performed on the significantly differentially expressed genes in these microglial cell lines at 3 h and 6 h post LPS-treatment likewise indicated a significant negative impact on the transcriptional activity of NFKB1 at the 6 h-time point (3 h, p value 0.078; 6 h, p value 0.0131) (Fig. 6b, c).

### Impairment of p65 NF-κB nuclear translocation in *Atg7* deficient microglia

**Fig. 7:**
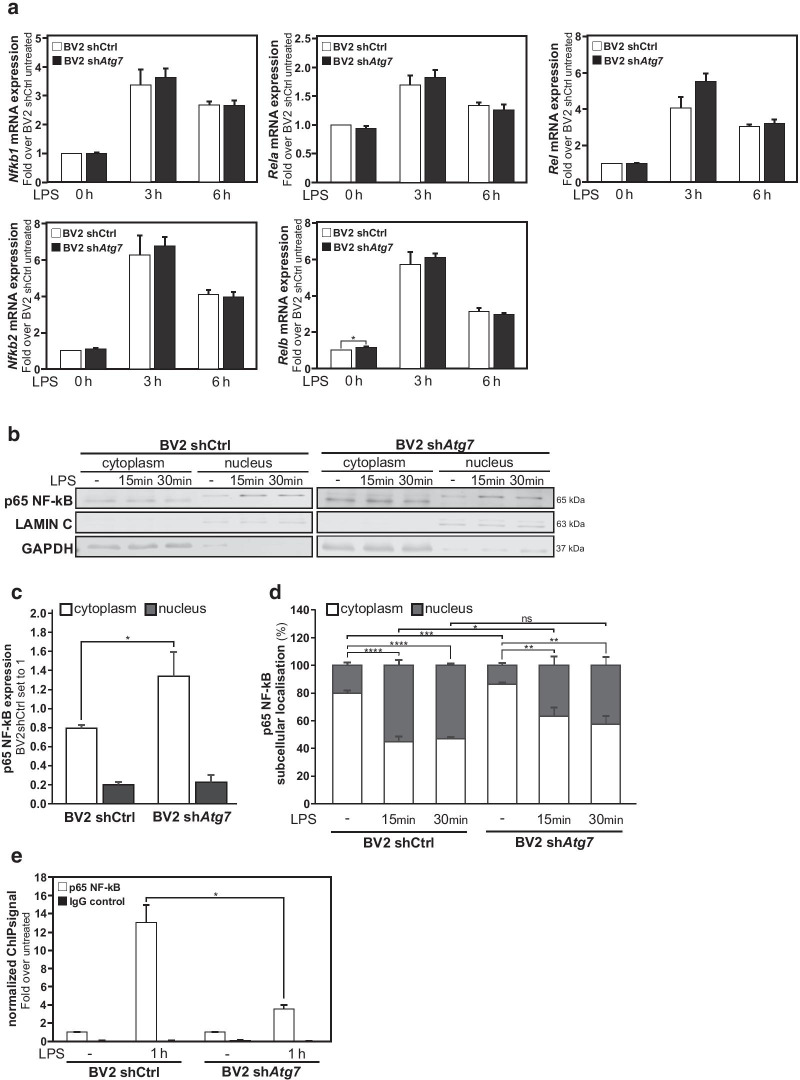
Impairment of p65 NF-κB nuclear translocation in *Atg7* deficient microglia upon LPS treatment. Microglial cells, i.e. sh*Atg7* and shCtrl BV2 cells were treated with LPS (100 ng/ml) for 15 min or 30 min. **a** Comparison of *Nfkb1, Nfkb2, Rel, Rel and Relb* (NF-κB family members) mRNA expression measured by RT-qPCR in sh*Atg7* and shCtrl BV2 cells. **b** Immunoblot analysis of p65 NF-κB catalytic subunit in cytoplasmic versus nuclear subcellular fractions in sh*Atg7* and shCtrl BV2 cells. The expression of LAMIN C and GAPDH were used as loading controls for cytoplasmic and nuclear fraction, respectively. **c** Graphs show the quantification for p65 NF-κB protein expression in cytoplasm and nucleus in untreated sh*Atg7* and shCtrl BV2 cells (total expression in both subcellular fractions set to 1, for shCtrl BV2 cells). **d** Graphs show the quantification of the percentual localization of total p65 NF-κB protein expression in cytoplasmic versus nuclear fractions in sh*Atg7* and shCtrl BV2 cells under each condition, i.e. untreated, 15 min or 30 min LPS-treatment. **e** ChIP analysis of p65 NF-κB enrichment at a *Nos2* promoter region encompassing a kB DNA binding site in sh*Atg7* and shCtrl BV2 cells treated with LPS (100 ng/ml) for 1 h. IgG was used as control. Values are a mean of 3 (**e**) 4 (**a**), or 5 (**c**, **d**) independent experiments ± SEM and considered significant for *p < 0.05, **p < 0.01, ***p < 0.001, ****p > 0.0001. n.s., not significant for the indicated comparison

Thereafter, we decided to gain insights into the possible mechanism behind the observed negative impact on NFKB1 transcriptional activity observed upon *Atg7* deficiency in microglia. First, the mRNA expression levels for the genes encoding the five subunits of the NFκB family, *Nfkb1*, *Nfkb2*, *Rela*, *Rel* and *Relb* were investigated by qRT-PCR in both untreated and 3 or 6 h LPS-treated sh*Atg7* BV2 and shCtrl BV2 microglia. Except for *Relb* at baseline level, the expression levels for the NF-κB subunits messengers were not found to be significantly differently expressed in the *Atg7*-deficient microglia as compared to the control microglia (Fig. 7a).

In the cytoplasm compartment, the p50 NF-κB subunit (encoded by *Nfkb1* gene) form a complex with the p65 NF-κB subunit (encoded by *Rela* gene) to generate the p65/p50 NF-κB heterodimers transcription factor, which upon translocation to the nucleus can exert transcriptional activities. In this p65/p50 dimer complex, only the p65 NF-κB subunit, contain a carboxy-terminal transactivation domains, responsible for the transcriptional activities of this NF-κB transcription factor (reviewed in [49]). Therefore, the cytoplasm to nucleus translocation of this catalytic active subunit of NF-κB, is used to assess the NF-κB potential transcriptional activity. In fact, subcellular fractionation (cytoplasmic versus nuclear fractions) and immunoblotting analysis revealed that the cytoplasm to nucleus translocation of the catalytic active subunit of NF-κB, p65, upon LPS stimulation is significantly impaired in sh*Atg7* BV2 cells as compared to shCtrl BV2 cells (Fig. 7b, d). This immunoblot analysis for p65, also confirmed at the protein level, that *Atg7*-deficiency in microglia was not associated with a reduction in the expression level of this catalytic active subunit of NF-κB. Instead, accumulation of the p65 NF-κB subunit was observed in the cytoplasmic fraction of sh*Atg7* BV2 cells as compared to shCtrl BV2 cells (Fig. 7b, c). Finally, p65 NF-κB subunit chromatin occupancy at a κB DNA binding site in the promoter region of the *Nos2* gene was investigated by chromatin immunoprecipitation (ChIP). This ChIP analysis demonstrated a reduced enrichment for this catalytic active subunit of NFκB, on a *Nos2* promoter region encompassing a NF-κB-responsive element in response to LPS treatment in sh*Atg7* BV2 microglia as compared to LPS-treated shCtrl BV2 microglia (Fig. 7e).

Collectively, these data demonstrated that a regulation of NF-κB family gene expression per se does not account for the observed reduction in NFKB1-dependent transcriptional activities observed in the *Atg7*-deficient microglia. Instead, it appears that the cytoplasm to nucleus translocation of the p65 catalytic active subunit containing NF-κB heterodimers, and chromatin recruitment upon LPS stimulation is impaired in the microglia deficient for *Atg7*.

### *Atg7* deficiency in microglia reduces the neurotoxicity associated with their response to pro-inflammatory stimulus

In summary, we report that deficiency in *Atg7* expression in BV2 microglia is associated with a lowered response to LPS treatment, a potent activator of the microglial pro-inflammatory phenotype, as illustrated by impaired induction of *Nos2* gene expression (Fig. 2a–c). In contrast, BV2 cells infected with lentivirus expressing a shRNA targeting *Atg7* expression, or with a control scramble shRNA used as control, showed no significant difference in their response to IL-4, as evaluated by the induction of *Arg1* gene expression (Fig. 2d–f). These striking differences observed in the molecular responses of these cells to the different stimuli lead us to investigate whether this translated into functional differences.

**Fig. 8:**
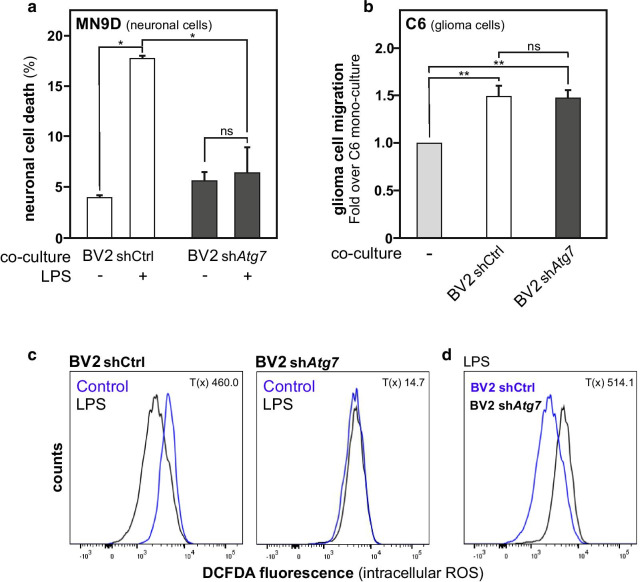
Microglial *Atg7* deficiency is associated with reduced neurotoxicity capability but does not alter tumor migration supporting function. **a** Neurotoxicity of LPS-treated BV2 cells on dopaminergic MN9D neuronal cells was assessed by co-culturing the neurons with either shCtrl or sh*Atg7* cells and subsequentially treating them with LPS (100 ng/ml) for 24 h. Neurons, stained with a cell tracker, with abnormal nucleus morphology were counted and displayed in the graph as total cell death in percent for each co-culture condition. **b** Quantification of the migration of C6 glioma cells in transwells with shCtrl or sh*Atg7* BV2 microglia. Results are relative compared to the C6 migration in wells without BV2 cells. **c** Logarithmic display of fluorescence representing intracellular ROS in shCtrl and sh*Atg7* cells after 1 h of LPS (100 ng/ml) as compared to unstimulated. **d** Intracellular ROS in shCtrl and sh*Atg7* cells after 1 h of LPS (100 ng/ml). Values are a mean of 3 (**a**, **b**) or 4 (**c**) independent experiments ± SEM and considered significant for *p < 0.05, **p < 0.01. n.s., not significant for the indicated comparison. **c**, **d** Chi square test is shown as statistical value to compare the cell populations in both graphs

Microglia activation towards a pro-inflammatory cell phenotype can trigger neurotoxicity. Indeed, these cells are a prominent source of pro-inflammatory factors and oxidative stress such as neurotoxic reactive oxygen species (ROS) [6, 52, 53]. Using microglial sh*Atg7* BV2 cells, or shCtrl BV2 cells, in co-culture with MN9D dopaminergic neurons, we examined whether the alteration in transcriptional program by selective knockdown of *Atg7* was associated with loss of microglial-mediated neurotoxicity. In agreement with earlier studies [6, 54], LPS treatment activated the shCtrl BV2 microglia and caused dopaminergic neurons to die. Importantly, we found the reduced LPS-induced microglia activation in sh*Atg7* BV2 resulted in lower numbers of cell death in the co-cultured dopaminergic neurons at the investigated time point (Fig. 8a and Additional file 1: Figure S5a, b).

IL-4 treatment is reported to promote the polarization of microglia towards a tumor-supportive phenotype that is observed in the context of brain tumors. In fact, microglia are attracted towards gliomas in large numbers, where the tumor cells shut down the inflammatory properties of microglia and modulate them to exert tumor-trophic functions [8, 10]. It is believed that glioma-derived molecules, including IL-4, contribute to the shift of glioma-infiltrating microglia towards the tumor-supportive phenotype [55]. Microglia can cause an increase in migratory functions of glioma cells. Therefore, C6 glioma cell migration capability was used as a biological functional readout to monitor the impact of *Atg7* deficiency on the response of BV2 microglia to a stimulus promoting an immunosuppressive and tumor-supportive phenotype. In agreement with the reported absence of an altered response to IL-4 treatment, sh*Atg7* BV2 cells and shCtrl BV2 cells were equally efficient in promoting C6 glioma cells migration (Fig. 8b and Additional file 1: Figure S5c). Release of oxidative stress factors by microglia, such as ROS, contribute to the cells’ neurotoxic capability. Hence, to further confirm the impact of *Atg7* deficiency on the pro-inflammatory phenotype and associated neurotoxicity in microglia, intracellular ROS was measured by flow cytometric analysis using the DCFDA ROS indicator. One-hour after LPS treatment, control microglial cells exhibited a robust drop in intracellular ROS, which was not observed in the *Atg7*-deficient microglia (Fig. 8c). Comparison of 1 h LPS-treated sh*Atg7* BV2 cells and shCtrl BV2 cells confirmed the *Atg7*-deficient microglia retained intracellular ROS as compared to the control ones (Fig. 8d). Thus, these data indicate that deficient *Atg7* gene expression in microglia leads to alterations in NF-κB-dependent expression of inflammation response-related genes. This selectively impacted their ability to respond to a pro-inflammatory stimulus, such as LPS, and in turn reduced their neurotoxicity.

## Discussion

The activation of microglia toward specific phenotypes exerting unique functions, could be considered as a feature of all brain related disorders, where these reactive immune cells are reported to contribute to the pathogenesis (reviewed in [3, 56, 57]). Consequently, targeting microglia as a therapeutic strategy to combat CNS related diseases, including neurodegenerative as well as proliferative disorders, has gained significant interest [58, 59]. The depletion of the microglial cell population has been reported to lead to neuroprotective effects by reducing neuroinflammation in various animal models of diseases (reviewed in [60]). Myeloid cells depend on the stimulation of the colony-stimulating factor-1 receptor (CSF-1R) for their survival, and therefor chemical inhibition of CSF-1R has been extensively used to deplete microglia [61]. Microglial depletion has also been considered for the treatment of brain neoplasms with some mitigated results due the acquisition of resistance to CSF-1R inhibition [62, 63].

Considering reports of acquired resistance to microglial inhibition, and the fact that even in the context of a diseased brain microglia may still be required for homeostatic functions, this global microglial depletion approach is being questioned in the field. Depletion and repopulation strategies are now being considered as an alternative strategy, in which enforced microglial repopulation could lead to replacement of dysfunctional cells, through new microglia that hold the potential to resolve an ongoing neuroinflammation [60]. A second strategy, closely associated to the topic of the current investigation, is based on the compelling evidence that the disease-associated microglia phenotypes are each characterized by unique gene expression profiles mediating their distinctive biological functions. From a therapeutic perspective, it would seem attractive to selectively target a subset of microglia exhibiting a disease-dependent reactive state, based on the modulation of their unique molecular signature. The so-called disease-associated microglia (DAM) identified in the context of AD, whose transcriptional program acquisition depends on the microglia-specific immunoreceptor TREM2 (triggering receptor expressed on myeloid cells 2)-dependent signaling, offer a promising example of modulation of a specific microglial phenotype [14, 23]. In a mouse model of AD that expresses five human familial AD gene mutations (5XFAD), knockdown of the *Trem2* gene led to an absence of microglia exhibiting the DAM phenotype [14]. Mechanistic target of rapamycin (mTOR) signaling is found to be upregulated in tumor-associated microglia in response to glioblastoma cell stimulation, as well as in microglia in response to a LPS challenge [64, 65]. Ras homolog enriched in brain 1 (Rheb1), an activator of the mechanistic target of rapamycin complex 1 (mTORC1), and thereby an mTOR-dependent signaling regulator, represents an additional regulator of microglial transcriptional programs. As recently reported, the genetic depletion of *Rheb1* increases transcription of inflammatory genes in microglia in the context of LPS stimulation [65], as well as in the tumor microenvironment situation [64].

Here, we report that even an incomplete silencing of *Atg7* gene expression in microglia can slow down the pro-inflammatory response of these cells induced by a challenge with LPS, a TLR4 ligand and potent inflammogen. Importantly, this diminished microglial pro-inflammatory response translated into reduced neurotoxicity, i.e. loss of biological function associated with this microglial reactive state.

The microglial cells used, i.e. sh*Atg7* BV2 cells, despite displaying a significant decrease (> 80% reduction) in *Atg7* gene expression, were still able to respond to autophagy stimuli. This suggests that the observed inhibition of the microglial pro-inflammatory response is not due to an abrogation of the autophagic process. Supporting this proposal, inhibition of autophagy with 3-methyladenine (3-MA), an inhibitor of phosphatidylinositol 3-kinases (PI3K) which is known to be essential for induction of this process, did not block but rather exacerbated LPS-induced pro-inflammatory responses in microglial N9 cells [44, 66]. Moreover, in our microglial model system, pre-treatment with BafA_1_, which blocks the autophagic flux by decreasing autophagosome–lysosome fusion, was not able to recapitulate the reduction in the pro-inflammatory response induced by *Atg7* depletion in BV2 microglia cells. In fact, as reported for the N9 microglia cells, chemical inhibition of autophagy exacerbated LPS-induced *Nos2* gene expression. Likewise, treatment with 3-MA or with chloroquine, which also blocks the autophagic flux by decreasing autophagosome–lysosome fusion, are not able to recapitulate the reduction in the pro-inflammatory response induced by *Atg7* depletion in endothelial cells [67, 68]. Likewise, in macrophages ATG7 was recently reported to be dispensable for the autophagic-related conjugation of LC3 with phosphatidylethanolamine (PE), i.e. formation of LC3-II observed upon induction of an inflammatory response by thioglycolate. Indeed, experiments performed with primary *Atg5*-deficient and *Atg7*-deficient macrophages, demonstrated that whereas ATG5 is required for the LC3 lipidation, ATG7 is dispensable for this process in these myeloid cells [69]. Collectively, these reports would argue that the observed effects on the pro-inflammatory response are due to the *Atg7* depletion and not an inhibition of autophagy per se.

Further, we report that *Atg7* deficiency in microglia is associated with significant transcriptomic alterations as compared to control ones, observed in the unstimulated cells and upon their stimulation with LPS. The *Atg7*-deficient microglia differentiated themselves in their gene expression profiles from the canonical ones, by reduced expression of genes grouped in GO terms like “immune response”, “stress response”, and “cytokine production”. A direct investigation of the transcriptomic data for genes reported to be associated with the response of microglia to a pro-inflammatory stimulus, further illustrated a possible alteration of their inflammatory response. Worth a note, the gene clusters and associated functions found to be decreased in the GO analysis of unstimulated and LPS-treated *Atg7*-deficient microglia shared similarity with the results found to be enriched in primary microglia isolated after 4 h from LPS injected mice [70]. Upon LPS stimulation, a reduction in the number of significantly differentially expressed genes between sh*Atg7* BV2 and shCtrl BV2 cells was observed. It can only be speculated about the reason for this reduction in gene expression profile differences. One hypothesis is that the two cell lines which exhibit remarkable transcriptome differences at baseline, despite significantly different kinetic in their response to LPS treatment tend to acquire overtime a microglial proinflammatory state of activation associated with a gene expression profile which then reflect into more similar transcriptomes. Transcription factors are essential for the selective regulation of gene expression profiles and the acquisition and maintenance of cell phenotypes. Transcriptional control of both microglia identity as well as their disease phenotypes is well recognized (reviewed in [12]). The transcriptomic profile of *Atg7*-deficient microglia revealed diminished expression of several genes encoding for transcription factors, including CCAAT enhancer binding proteins beta and delta (*Cebpd, Cebpb*), Interferon regulatory factor 7 (*Irf7*), Jun, JunB and JunD proto-oncogene (*Jun, Junb, Jund*) with FC < -1.5 and FDR < 0.05. However, even if the expression level of transcription factors may provide with an indication of their potential transcriptional activities in cells, accurate assessment includes the analysis of their regulon, i.e. the co-expression of their target genes [71]. The subsequent transcriptional regulatory networks analysis of the transcriptomic data obtained with *Atg7*-deficient microglia exposed that the NFKB1-dependent regulon was negatively affected, implying an impairment of the NF-κB-dependent signaling pathways in these cells. The transcriptional activity of NF-κB complexes is regulated by interaction with regulatory proteins, such as the inhibitor of NF-κB proteins (IκBs) [50]. Bcl-3, an atypical IκB, reported to bind to NF-κB p50 and p52 subunits and most likely provide transcriptional activation properties, was also found to be significantly reduced in *Atg7*-deficient microglia [72]. It should also be noted that *Cebpb* and *Junb* genes, whose expression were found to be reduced in *Atg7*-deficient microglia, belong to the NFKB1-dependent regulon. This could suggest that the loss in gene expression observed could be the result of the direct or indirect effect of reduced NF-κB transcriptional regulatory function. In fact, NF-κB can regulate the transcription of genes encoding for chemokines, cytokines, pro-inflammatory enzymes, pro-inflammatory transcription factors, and other factors to regulate the inflammatory reaction of microglia. Furthermore, the list of reports suggesting an inhibition of NF-κB as an efficient strategy to reduce a neuroinflammatory response in microglia, including BV2 microglia exposed to a LPS challenge, is extensive and expanding [73–78]. Members of the NF-κB family, activator protein-1 (AP-1) family including the Jun-related transcription factors, Interferon Regulatory Factor (IRF) family and CCATT-enhancer-binding proteins (C/EBP) family are all reported to regulate the expression of pro-inflammatory mediators in myeloid cells [12, 79].

Finally, a striking finding of the current investigation is that knockdown of microglial *Atg7* impacted negatively on the response to a pro-inflammatory challenge, resulting in reduced neurotoxicity. However, their response to the anti-inflammatory cytokine IL-4 was unaffected. Likewise, as compared to control microglia, the *Atg7*-deficient microglia did not display any difference in the glioblastoma-induced tumor-supportive function. Whereas transcriptional regulators have been proposed for selected disease-associated microglia phenotypes (e.g. TREM2 for DAM), *Atg7* would be the first candidate regulator of microglial transcriptome for selected microglial activation states.

## Conclusions

Microglia are CNS resident immune reactive cells that, due to their transcriptomic plasticity, can acquire various reactive states with unique biological functions. It is of relevant therapeutical significance to understand how to target a specific transcriptional program controlling a distinct microglial phenotype that might be dysfunctional or exacerbated in the context of a brain disease. Here, we identify *Atg7* as a selective modulator of an NF-κB-dependent transcriptional program controlling the pro-inflammatory response of microglia and uncover that microglial *Atg7* deficiency was associated with reduced microglia-mediated neurotoxicity upon inflammogen stimulation. The identification of distinct regulators controlling specific microglial transcriptional programs and thereby unique functions could lead to the development of novel therapeutic strategies aiming at manipulating selected microglial phenotypes instead of the whole microglial population with its associated pitfalls.

## Supplementary Information

**Additional file 1: Figure S1.** Reduction in *Atg7* expression decreases the ability of BV2 microglia to undergo autophagy. a) Immunoblot analysis and b) quantification of LC3-I and LC3-II expression versus ACTB in shCtrl BV2 cells versus sh*Atg7* BV2 cells. Cells were cultured in EBSS medium to induce starvation, or stimulated with 250 nM Torin1 for 2 h, to induce autophagy. Treatment with BafA_1_ (40 nM), late inhibitor of autophagy, before sample collection was used to block the autophagic flux. c) Comparison of *Nos2* mRNA expression measured by RT-qPCR in sh*Atg7* and shCtrl BV2 cells. Cells were pre-treated with BafA_1_ (40 nM) for 30 min and then treated with LPS (100 ng/ml) for 3 h. Values are a mean of 3 (c) or 4 (b) independent experiments ± SEM and considered significant for *p < 0.05, **p < 0.01. n.s., not significant for the indicated comparison. **Figure S2.** Venn diagram comparing number of differentially expressed genes 3 h and 6 h LPS treated control and Atg7-deficient microglia. Analysis performed to compare the number of significantly differentially expressed genes in the 3 h and 6 h LPS-treated sh*Atg7* BV2 microglia and shCtrl BV2 microglia, as compared to their respective untreated control (fold changes over control samples being used in these analysis). Details including the gene lists for each section are available in Additional file 4. **Figure S3.** Gene Ontology network analysis of differentially expressed genes found in GSEA dataset C7: immunologic signature gene sets. a–d) Network analysis of enriched GO term clusters generated from a gene list of overlap between the GSEA C7 dataset and significant (FDR < 0.05) differentially expressed genes comparing sh*Atg7* BV2 and shCtrl BV2. Nodes represent GO terms, clusters are nodes grouped based on similarity. Node size corresponds to number of genes. Node color corresponds to the significance of correlation, where the darker the color is, the more significant the FDR values. Lines represent the number of genes overlapping between nodes. Enrichment is shown for (a) less expressed genes in sh*Atg7* in untreated condition, (b) higher expressed genes in sh*Atg7* BV2 in untreated condition, (c) less expressed genes in sh*Atg7* after 3 h LPS (100 ng/ml) or (d) less expressed genes in sh*Atg7* after 6 h LPS (100 ng/ml), each compared to the respective control of shCtrl BV2. Details including the GO term clusters and overlapping gene lists for each condition are available in Additional file 5. **Figure S4.** Decreased *Apoe*/APOE expression in *Atg7* deficient microglia. Microglial cells, i.e. sh*Atg7* and shCtrl BV2 cells were treated with LPS (100 ng/ml) for 3 h or 6 h. a) Immunoblot analysis of APOE protein expression in sh*Atg7* and shCtrl BV2 cells. The expression of ACTB as housekeeping gene was used as loading control. Cells were treated with LPS (100 ng/ml) for 3 h or 6 h. b) Graphs show the quantification for APOE versus ACTB protein expression and in sh*Atg7* and shCtrl BV2 cells. Values are in comparison to untreated shCtrl BV2. c) Comparison of *Apoe* mRNA expression measured by RT-qPCR in sh*Atg7* and shCtrl BV2 cells. Values are in comparison to untreated shCtrl BV2. All values are a mean of 4 independent experiments ± SEM and considered significant for **p < 0.01. **Figure S5.** LPS-treatment does not increase cell death of MN9D neuronal cells. a) Cell death was assessed in dopaminergic MN9D neuronal cells treated with LPS (100 ng/ml) for 24 h. Neurons, stained with a cell tracker, with abnormal Hoechst-counterstained nucleus morphology were counted and displayed in the graph as total cell death in percent. b) Representative images of neurotoxicity assay described in Fig. 8 panel a (scale bar 20 µM). c) Representative images of the migration of DAPI-stained C6 glioma cells in transwells with shCtrl BV2 microglia or sh*Atg7* BV2 microglia. Medium alone in the lower transwell compartment was used as control (quantification depicted in Fig. 8 panel b). f Values are a mean of 3 independent experiments ± SEM. n.s., not significant for the indicated comparison.

**Additional file 2.** DEG lists and KEGG pathways comparing shCtrl BV2 cells with sh*Atg7* BV2 cells.

**Additional file 3.** DEG lists and KEGG pathways comparing LPS-treated sh*Atg7* BV2 with untreated sh*Atg7* BV2 cellsdat.

**Additional file 4.** Venn diagram gene lists and Metascape GO term clusters.

**Additional file 5.** Metascape GO term clusters for overlap with C7: immunologic signature genes.

## Data Availability

All data generated or analyzed during this study are included in this published article and its Additional information files.

## Abbreviations

ACTB: β Actin
AD: Alzheimer’s disease
APOE: Apolipoprotein E
ARG1: Arginase 1
ATG7: Autophagy related 7
BafA_1_: Bafilomycin A1
ChIP: Chromatin immunoprecipitation
GSEA: Gene set enrichment analysis
CSF-1R: Colony-stimulating factor-1 receptor
DAM: Disease-associated microglia
FOXO1: Forkhead box protein O1
GO: Gene ontology
IκB: Inhibitor of NF-κB protein
IL-4: Interleukin 4
LPS: Lipopolysaccharide
3-MA: 3-Methyladenine
mTOR: Mechanistic target of rapamycin
NF-κB: Nuclear factor of kappa B
NFKB1: Nuclear factor of kappa B subunit 1
NFKB2: Nuclear factor of kappa B Subunit 2
NOS2: Nitric oxide synthase 2
Rheb1: Ras homolog enriched in brain 1
ROS: Reactive oxygen species
shRNA: Small hairpin RNA
TLR4: Toll-like receptor 4
TREM2: Triggering receptor expressed on myeloid cells 2
