# Supplementary material for: Atg7 deficiency in microglia drives an altered transcriptomic profile associated with an impaired neuroinflammatory response
Source: Mol Brain. 2021 Jun 3;14:87. doi: 10.1186/s13041-021-00794-7 (PMC8173846; doi:10.1186/s13041-021-00794-7)
Supplement: Supplementary file 1 — Additional file 1: Figure S1. Reduction in Atg7 expression decreases the ability of BV2 microglia to undergo autophagy. a) Immunoblot analysis and b) quantification of LC3-I and LC3-II expression versus ACTB in shCtrl BV2 cells versus shAtg7 BV2 cells. Cells were cultured in EBSS medium to induce starvation, or stimulated with 250 nM Torin1 for 2 h, to induce autophagy. Treatment with BafA1 (40 nM), late inhibitor of autophagy, before sample collection was used to block the autophagic flux. c) Comparison of Nos2 mRNA expression measured by RT-qPCR in shAtg7 and shCtrl BV2 cells. Cells were pre-treated with BafA1 (40 nM) for 30 min and then treated with LPS (100 ng/ml) for 3 h. Values are a mean of 3 (c) or 4 (b) independent experiments ± SEM and considered significant for *p < 0.05, **p < 0.01. n.s., not significant for the indicated comparison. Figure S2. Venn diagram comparing number of differentially expressed genes 3 h and 6 h LPS treated control and Atg7-deficient microglia. Analysis performed to compare the number of significantly differentially expressed genes in the 3 h and 6 h LPS-treated shAtg7 BV2 microglia and shCtrl BV2 microglia, as compared to their respective untreated control (fold changes over control samples being used in these analysis). Details including the gene lists for each section are available in Additional file 4. Figure S3. Gene Ontology network analysis of differentially expressed genes found in GSEA dataset C7: immunologic signature gene sets. a–d) Network analysis of enriched GO term clusters generated from a gene list of overlap between the GSEA C7 dataset and significant (FDR < 0.05) differentially expressed genes comparing shAtg7 BV2 and shCtrl BV2. Nodes represent GO terms, clusters are nodes grouped based on similarity. Node size corresponds to number of genes. Node color corresponds to the significance of correlation, where the darker the color is, the more significant the FDR values. Lines represent the number of genes overlapp [file 13041_2021_794_MOESM1_ESM.pdf]

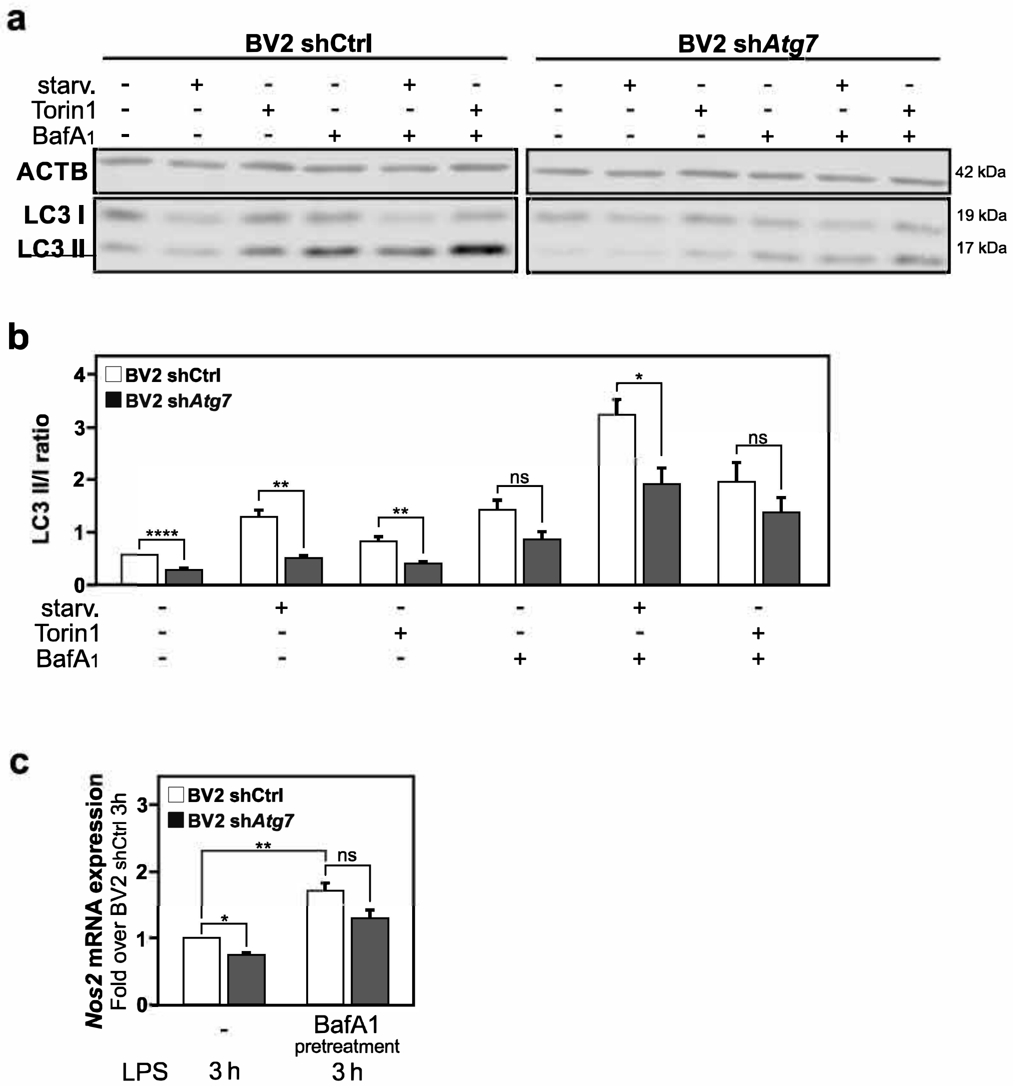

Figure S1 Friess *et al*.

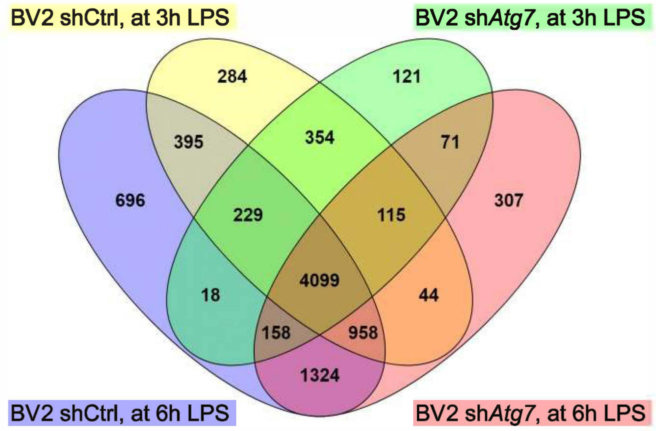

Figure S2 Friess *et al*.

a

Cytoscape network of genes overlapping GSEA C7 list
Less expressed in BV2 sh*Atg7*, untreated

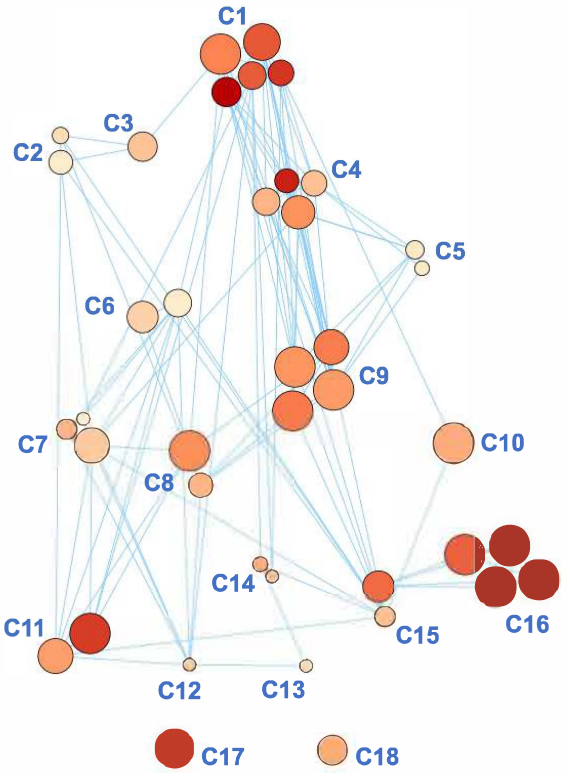

C1 CD4-positive, alpha-beta T cell activation
C2 collagen biosynthetic process
C3 cell-substrate adhesion
C4 negative regulation of immune effector process
C5 regulation of interferon-beta production
C6 neuron apoptotic process
C7 modulation of process of other organism
C8 positive regulation of locomotion
C9 inflammatory response
C10 apoptotic signaling pathway
C11 blood vessel morphogenesis
C12 positive regulation of vascular permeability
C13 positive regulation of histone deacetylation
C14 regulation of pre-miRNA transcription by RNA polymerase II
C15 dendrite development
C16 regulation of plasma membrane bounded cell projection organization
C17 small GTPase mediated signal transduction
C18 maintenance of location

b

Cytoscape network of genes overlapping GSEA C7 list
More expressed in BV2 sh*Atg7*, untreated

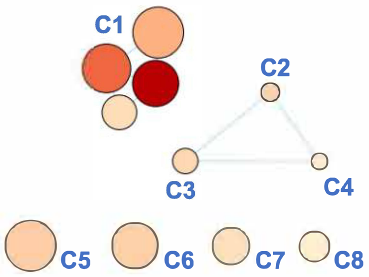

C1 regulation of translation
C2 somatic recombination of immunoglobulin gene segments
C3 maintenance of cell number
C4 chromatin silencing at telomere
C5 cell cycle
C6 protin ubiquitination
C7 establishment of protein localiszation to organelle
C8 ribosome biogenesis

c

Cytoscape network of genes overlapping GSEA C7 list
Less expressed in BV2 sh*Atg7*, 3h LPS

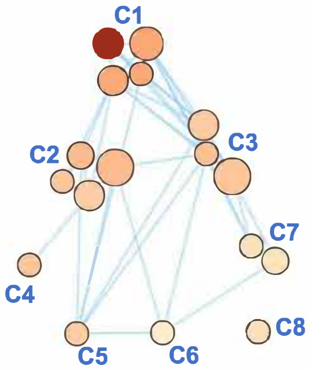

C1 CD4-positive, alpha-beta T cell activation
C2 regulation of epithelial cell migration
C3 regulation of epithelial cell differentiation
C4 endochondral bone growth
C5 vasculogenesis
C6 placenta development
C7 fat cell differentiation
C8 response to interferon-gamma

d

Cytoscape network of genes overlapping GSEA C7 list
Less expressed in BV2 sh*Atg7*, 6h LPS

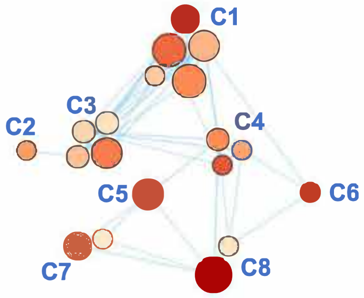

C1 cytokine-mediated signaling pathway
C2 hyaluronan metabolic process
C3 apoptotic signaling pathway
C4 collagen biosynthetic process
C5 cell projection assembly
C6 cellular response to interferon-gamma
C7 Cdc42 protein signal transduction
C8 actin cytoskeleton organization

Figure S3 Friess *et al.*

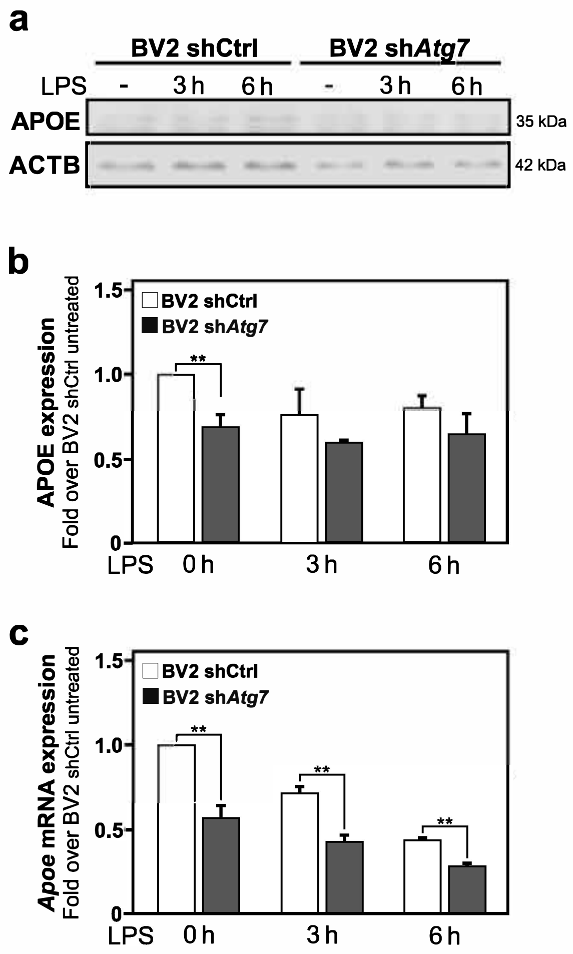

Figure S4 Friess *et al.*

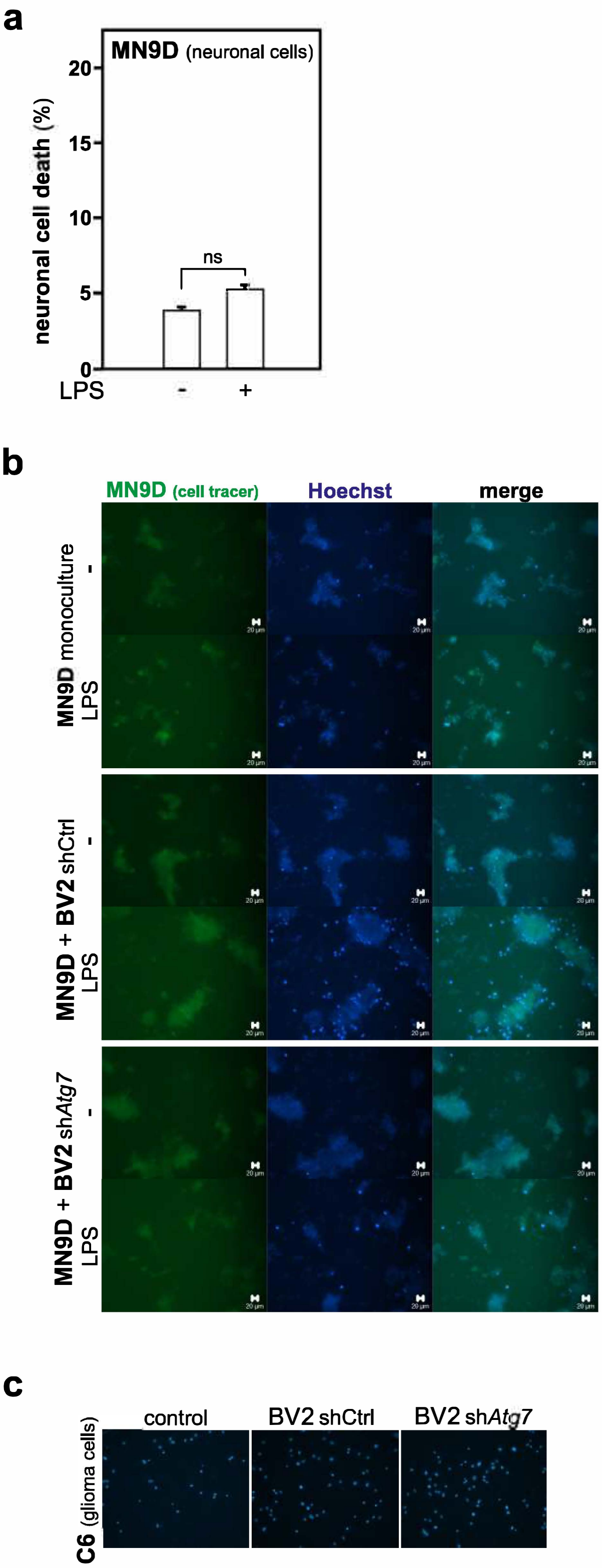

Figure S5 Friess *et al.*
